# How Much Lox Is a Grizzly Bear Worth?

**DOI:** 10.1371/journal.pbio.1001304

**Published:** 2012-04-10

**Authors:** Jonathan Chase

**Affiliations:** Freelance Science Writer, Saint Louis, Missouri, United States of America

## Abstract

Using grizzly bears as surrogates for “salmon ecosystem” function, the authors develop a generalizable ecosystem-based management framework that enables decision makers to quantify ecosystem-harvest tradeoffs between wild and human recipients of natural resources like fish.

**Figure:**
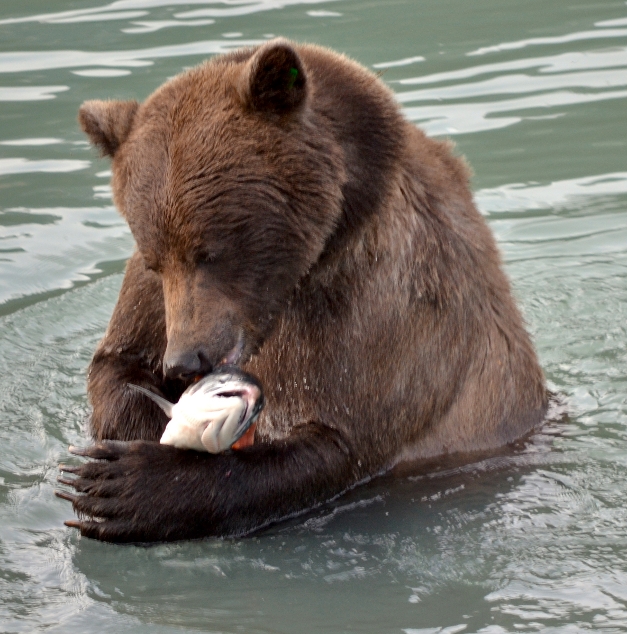
Wild salmon are valued as a sustainable seafood option for humans, but can we consider how much salmon bears and ecosystems need when setting management goals? Image credit: Jennifer Allen.

Yogi Bear, the ursine protagonist of a classic American cartoon, was doggedly persistent in his quest to pilfer picnic baskets from unsuspecting human visitors to the fictitious Jellystone Park. Because bears and humans are among the most omnivorous in the animal kingdom, it's not surprising that we often find ourselves competing for the same food. While we often think of this interaction as one-sided, with bears like the fictional Yogi taking food from people, in the global picture, humans have in fact taken much more food out of the mouths of bears than the other way around.

North American Pacific salmon fisheries provide an excellent example of how humans usurp resources that have provided grizzly bears, as well as many other species on sea and land, with food for eons. As the fish spend most of their lives in the sea only to head back to their natal streams to breed and die, they transport a large amount of marine-derived nutrients to bears and other species in those stream ecosystems. Even terrestrial plants get in on the action; the fish carcasses can provide up to a quarter of the nitrogen budget for plants near these streams. “Thus, the losses to the ecosystem associated with salmon harvesting by people can be quite high.”

Today's sustainability-conscious consumer cares not only whether the seafood they are eating was harvested to maintain sustainable populations, but also whether there are incidental consequences of the harvest, such as bycatch of threatened species or unintended detriment to the broader ecosystem. Ecosystem-based fisheries management (EBFM) is an emerging paradigm that endeavors to capture both the ecological and economic costs and benefits when valuing fisheries harvest and making management decisions. To date, however, scientists and managers applying EBFM have not been able to adequately compare those costs and benefits on the same playing field.

This is where the bears and salmon come in. In a new study published in *PLoS Biology*, Taal Levi, Chris Darimont, and colleagues devise a way to compare apples and oranges—or bears and salmon, as the case may be—so that any benefits to the bears, and by extension, the ecosystem, that arise from allowing more salmon to escape the fishermen's nets can be directly compared with the economic costs of lost harvest.

The authors first make the case that understanding how salmon influence grizzly bear populations can provide a good proxy for how salmon influence the entire ecosystem. To do so, they used published data on the stable isotope ratios of carbon and nitrogen (^12^C to ^13^C and ^14^N to ^15^N) in bears (measured in their hair) from populations with different levels of access to salmon, and showed that the proportion of salmon in the diet of grizzlies was strongly correlated with the numbers of salmon in the streams. They then used the published relationship between the proportion of salmon in the diet of grizzlies and their increased fitness and population growth to link salmon numbers with bear numbers.

With these correlations in hand, the authors devise a straightforward model by which fisheries management can be directly tied to grizzly bear success as a proxy for ecosystem-level benefits. Focusing specifically on runs of sockeye salmon (*Oncorhynchus nerka*), a highly regulated fishery of considerable economic and ecological importance, the model was used to examine how both fisheries and ecosystems should be influenced by letting more salmon pass through the fishermen's nets and head upstream—termed “escapement”.

For four coastal stocks of sockeye that breed in streams with other species of salmon (e.g., pink, chum), the models showed that allowing more sockeye escapement usually led to a “win-win”; higher long-term yield for fisheries and more nourishment for bears and ecosystems. However, a trade-off between economic and ecosystem goals emerged in two inland catchments where runs of salmon other than sockeye were small. By estimating the expected economic losses to these fisheries that would be required to maintain grizzly populations (around US$500,000–$700,000 per stock each year), this framework provides a more informed way to manage the balance between sustainable economies and ecosystems.

Rarely do sustainable economies and sustainable ecosystems result from the same policies. As we approach another presidential election in the US, you can be certain we'll be hearing much politically charged rhetoric to that effect. Because economic and environmental systems are typically quantified in very different ways, we have not had the ability to directly compare how harvesting policies influence the economy and the ecosystem in the same terms. Although the study by Levi, Darimont, and colleagues focused on the use of salmon by people and bears, it points the way towards devising a more direct means by which to compare fisheries management options for both economic and ecological outcomes, and to make more informed decisions as a result.

**Levi T, Darimont CT, MacDuffee M, Mangel M, Paquet P, et al. (2012) Using Grizzly Bears to Assess Harvest-Ecosystem Tradeoffs in Salmon Fisheries. doi: 10.1371/journal.pbio.1001303**

